# Human Hepatocyte Growth Factor (hHGF)-Modified Hepatic Oval Cells Improve Liver Transplant Survival

**DOI:** 10.1371/journal.pone.0044805

**Published:** 2012-09-18

**Authors:** Zhu Li, Juan Chen, Li Li, Jiang-Hua Ran, Xue-Hua Li, Zhi-Heng Liu, Gui-Jie Liu, Yan-Chao Gao, Xue-Li Zhang, Hiu-Dong Sun

**Affiliations:** 1 Department of Hepatobiliary Surgery, Liaocheng People's Hospital, Liaocheng, Shandong, China; 2 The First People's Hospital of Kunming, Kunming, Yunnan, China; University of Colorado School of Medicine, United States of America

## Abstract

Despite progress in the field of immunosuppression, acute rejection is still a common postoperative complication following liver transplantation. This study aims to investigate the capacity of the human hepatocyte growth factor (hHGF) in modifying hepatic oval cells (HOCs) administered simultaneously with orthotopic liver transplantation as a means of improving graft survival. HOCs were activated and isolated using a modified 2-acetylaminofluorene/partial hepatectomy (2-AAF/PH) model in male Lewis rats. A HOC line stably expressing the HGF gene was established following stable transfection of the pBLAST2-hHGF plasmid. Our results demonstrated that hHGF-modified HOCs could efficiently differentiate into hepatocytes and bile duct epithelial cells *in vitro*. Administration of HOCs at the time of liver transplantation induced a wider distribution of SRY-positive donor cells in liver tissues. Administration of hHGF-HOC at the time of transplantation remarkably prolonged the median survival time and improved liver function for recipients compared to these parameters in the other treatment groups (*P*<0.05). Moreover, hHGF-HOC administration at the time of liver transplantation significantly suppressed elevation of interleukin-2 (IL-2), tumor necrosis factor-α (TNF-α) and interferon-γ (IFN-γ) levels while increasing the production of IL-10 and TGF-β1 (*P*<0.05). HOC or hHGF-HOC administration promoted cell proliferation, reduced cell apoptosis, and decreased liver allograft rejection rates. Furthermore, hHGF-modified HOCs more efficiently reduced acute allograft rejection (*P*<0.05 versus HOC transplantation only). Our results indicate that the combination of hHGF-modified HOCs with liver transplantation decreased host anti-graft immune responses resulting in a reduction of allograft rejection rates and prolonging graft survival in recipient rats. This suggests that HOC-based cell transplantation therapies can be developed as a means of treating severe liver injuries.

## Introduction

Orthotopic liver transplantation is believed to be the only definitive therapy for patients with end-stage liver diseases; however, severe complications such as acute allograft rejection commonly occur [1], [2]. Transplantation of stem cells or progenitor cells, including hepatocytes and hepatic oval cells (HOCs) that have self-renewal and differentiation potential have been utilized as alternative approaches for mediating repair of damaged tissue resulting in liver regeneration [3], [4], [5]. Nevertheless, loss of transplanted donor cells due to rejection (or other mechanisms) seems to be the major limitation for broader application of liver cell therapy [6]. Therefore, improvements to the efficacy of donor cells and prevention of rejection, is needed as a means of improving transplanted liver survival rates.

Hepatocyte growth factor (HGF) is a multifunctional growth factor which prevents apoptosis, invasion of extracellular matrices, and proliferation [7]. Moreover, it has been demonstrated that the HGF/c-met signaling pathway is required for efficient liver regeneration and repair [8]. Previous studies showed that adenovirus-mediated HGF gene expression could regulate HOC proliferation [9]. In addition, accumulating evidence indicates that HGF can accelerate this proliferation and possibly promote HOC differentiation in the 2-acetylaminofluorene/partial hepatectomy (2-AAF/PH) rat model [10], [11]. Moreover, it has been demonstrated that HGF can prevent the development of chronic allograft nephropathy in rats [12]. In a mouse model of cardiac transplantation, HGF suppressed acute and chronic allograft rejection [13]. Based on this evidence, we hypothesized that overexpression of HGF in HOCs may serve to promote graft survival by inhibiting allograft rejection after orthotopic liver transplantation.

In the present study, we investigated the potential effects of combined hHGF-modified HOCs and liver transplantation on the improvement of acute allograft rejection and recipient survival in rats. Our findings suggest that hHGF-modified HOC transplantation may provide a valuable approach for the treatment of patients with severe liver damage.

## Results

### hHGF-modified HOC characteristics

**Figure 1:**
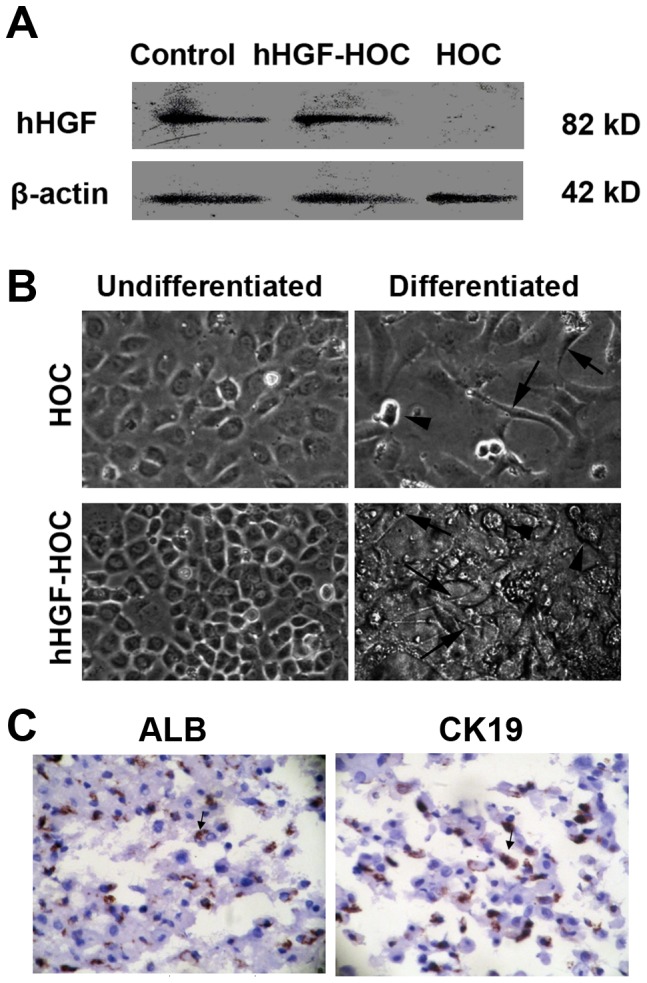
hHGF-modified HOC differentiation potential. LIF was withdrawn from culture medium to induce cell differentiation. (A) Intracellular hHGF protein expression was determined by Western blot analysis. hHGF was used as positive control and β-actin used as an internal control. (B) Undifferentiated or differentiated normal HOCs or hHGF-modified HOCs were monitored under phase contrast microscopy. Arrows indicated long-spindle like bile duct epithelial cells and arrowheads indicat the large, round-shaped hepatocytes. (C) Immunostaining of differentiated cells with specific antibodies for ALB or CK19. Arrows indicated immuno-positive cells (dark brown cells). Sections were double-stained with H&E. Magnification ×200.

Enhanced hHGF expression in hHGF-modified HOCs was assessed by Western blot analysis (Fig. 1A). Using ELISA-based assays we detected significantly increased levels of hHGF expression in medium collected from hHGF-modified HOC cultures compared to medium from untreated HOCs (7.324±0.477 ng/ml vs. 0 ng/ml). Unpublished work from our laboratory showed that HOCs modified to express hHGF promoted proliferation, and HOCs stably expressing the hHGF efficiently differentiated into large round-shaped hepatocytes and long, spindle-like bile duct epithelial cells *in vitro* with most HOCs differentiating into bile duct epithelial cells (Fig. 1B). RT-PCR analysis demonstrated that both undifferentiated and differentiated hHGF-HOCs expressed the liver and bile duct markers ALB and CK19 (Table 1). In addition, CK19 mRNA levels in undifferentiated hHGF-HOCs were significantly lower than that in differentiated hHGF-HOCs (*P*<0.05). Immunostaining with specific antibodies for ALB or CK19 further showed the presence of immuno-positive cells in differentiated hHGF-HOCs (Fig. 1C).

**Table 1:** Quantification of ALB and CK19 mRNA levels using quantitative RT-PCR.

	ALB	CK19
Liver	−1.127±0.486* #	−1.829±0.624* #
Bile duct	−6.534±0.489* #	−1.753±0.692*
Undifferentiated hHGF-HOCs	−1.814±0.603	−3.792±0.953
Differentiated hHGF-HOCs	−1.773±0.538	−1.476±0.615*

Data were quantified from 20 independent experiments and normalized with β-actin. Differences were analyzed using an analysis of variance or an independent sample *t*-test. Negative logs were calculated as described in the Materials and Methods.

* *P*<0.05 *vs.* undifferentiated hHGF-HOCs;

# *P*<0.05 *vs.* differentiated hHGF-HOCs.

### Liver transplantation combined with HOC administration

**Figure 2:**
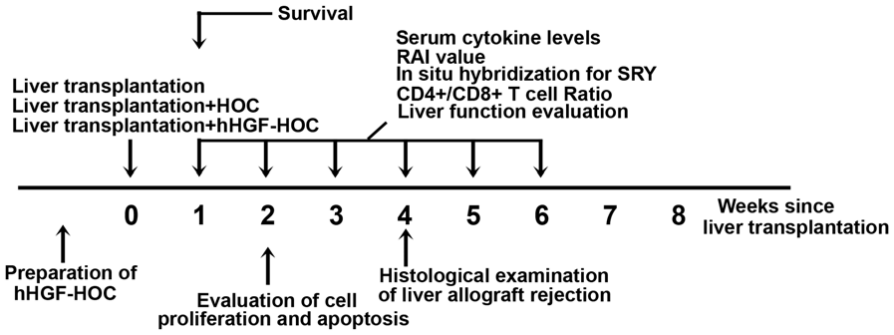
Experimental design outline.

**Figure 3:**
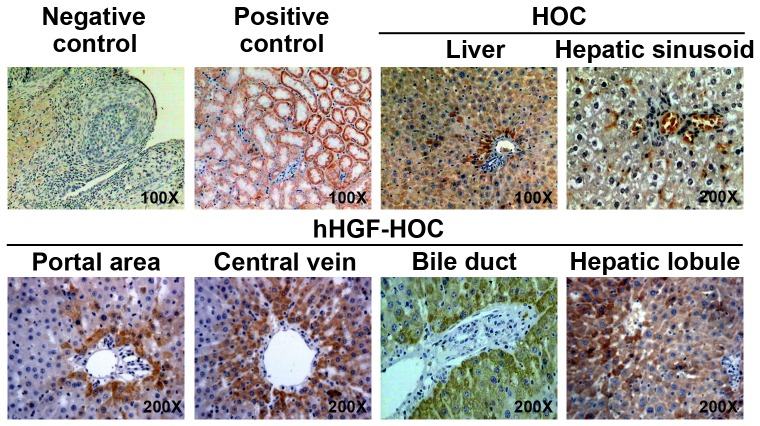
*In situ* hybridization of SRY-positive donor HOCs. *In situ* hybridization was performed 21 days post transplantation. Ovarian tissue was used as a negative control and renal tissues from male Lewis rats used as positive controls. SRY-positive cells exhibited dark brown staining of their nuclei and/or cytoplasm. Following HOC administration to liver transplant recipients, SRY-positive cells were found in liver tissues and hepatic sinusoids. Additionally, SRY-positive hHGF-HOCs were observed in the surrounding portal area, central vein and bile duct, and were diffusely distributed in hepatic lobules. Magnification is indicated on each image.

To determine the *in vivo* effects of hHGF-modified HOCs, cells were transplanted into female Lewis rats that also received an orthotopic liver transplant. The outline of the experimental design is presented in Fig. 2. In order to evaluate cell transplantation efficacy, expression of the Y-chromosome-specific SRY gene (present only on male donor HOCs) was assessed by *in situ* hybridization. Following transplantation and administration of untreated HOCs, SRY-positive donor HOCs infiltrated the portal area (Fig. 3). Apoptotic or dead SRY-positive cells were also found in hepatic sinusoids. Compared to the HOC group, hHGF-HOC transplantation induced a wider distribution of donor cells; that is, SRY-positive cells were observed surrounding the portal area, central vein, bile duct, and were diffusely distributed in hepatic lobules (Fig. 3). The percentage of SRY-positive cells was greatly increased over time post transplantation in transplanted rats also treated with hHGF-HOCs compared to transplant rats treated with HOC only (*P*<0.05, Table 2). Moreover, peripheral hHGF levels were somewhat upregulated in hHGF-HOC treated transplant animals (*P*<0.05 compared to other groups, Table 2). Sixteen days post transplantation, gross examination of the control group livers (orthotopic liver transplantation without cell transplantation) revealed that swelling and cirrhosis were accompanied by liver ischemia, congestion and hemorrhage. Localized congestion without cirrhosis or necrosis was observed in HOC-treated, liver transplant rats. In contrast, liver transplant rats treated with hHGF-modified HOCs presented with normal liver morphology.

**Table 2:** Measurement of parameters associated with HOC administration and liver transplantation.

	Weeks since liver transplantation					
	1	2	3	4	5	6
**Percentage of SRY-positive cells (%)**						
HOC+liver transplantation	14.0±3.7*	18.1±3.4*	21.9±3.7*	29.0±5.5*	31.4±5.9*	30.6±3.8*
hHGF-HOC+liver transplantation	20.9±7.7	33.5±6.8	41.3±4.9	59.7±11.3	63.5±5.1	64.7±8.4
**Peripheral hHGF level (ng/ml)**						
Liver transplantation	0*	0*	0*	0*	0*	0*
HOC+liver transplantation	0*	0*	0*	0*	0*	0*
hHGF-HOC+liver transplantation	0.80±0.38	1.78±0.59	2.78±0.76	3.88±0.79	4.98±0.68	5.74±0.45
**CD4^+^/CD8^+^ T cell Ratio**						
Liver transplantation	1.1±0.4	1.8±0.5* #	3.1±0.5* #	4.8±0.9* #	NA	NA
HOC+liver transplantation	1.0±0.3	1.4±0.2*	1.8±0.3*	2.1±0.3*	2.4±0.4*	2.3±0.5*
hHGF-HOC+liver transplantation	0.9±0.5	1.1±0.2	1.4±0.4	1.7±0.5	1.6±0.2	1.5±0.3
**ALT level (U/l)**						
Liver transplantation	66±19	220±147* #	680±396* #	968±203* #	NA	NA
HOC+liver transplantation	61±15	96±17	121±25	127±19	122±23*	105±32*
hHGF-HOC+liver transplantation	64±21	110±20	115±21	96±27	65±28	41±20
**DBil level (µM)**						
Liver transplantation	9.4±2.0	22.0±7.0* #	58.6±21.2* #	80.0±21.8* #	NA	NA
HOC+liver transplantation	8.3±3.0	12.2±3.5	19.8±8.2*	22.1±7.9*	25.3±5.5*	21.1±6.2*
hHGF-HOC+liver transplantation	8.6±2.1	11.1±3.3	12.8±1.3	12.8±2.7	13.6±1.7	11.3±3.6
**ALB level (g/l)**						
Liver transplantation	11.7±3.7*	11.3±2.2* #	9.1±2.5* #	8.2±1.6* #	NA	NA
HOC+liver transplantation	13.3±3.4	13.4±3.3*	14.9±1.9*	14.8±2.4*	15.9±2.4*	15.3±3.4*
hHGF-HOC+liver transplantation	14.5±3.6	16.8±4.1	20.6±3.8	21.0±2.4	23.4±1.5	23.6±1.9
**GGT level (U/l)**						
Liver transplantation	7.5±3.0	42.2±17.7* #	106±35* #	355±165* #	NA	NA
HOC+liver transplantation	6.2±3.9	24.4±12.0*	36.0±5.1*	65.9±14.4*	70.7±19.4*	71.5±16.6*
hHGF-HOC+liver transplantation	7.0±3.7	16.7±3.3	21.3±8.7	26.5±6.9	25.0±4.4	26.4±8.5
**ALP level (U/l)**						
Liver transplantation	95±18 295±158	295±158* # 295±158	258±171* # 295±158	680±190* # 295±158	NA	NA
HOC+liver transplantation	88±14	180±41*	196±40*	256±21*	221±41*	209±51*
hHGF-HOC+liver transplantation	93±16	130±46	133±17	100±33	105±17	86±26
**ChE level (U/l)**						
Liver transplantation	1735±525	1458±255	819±175* #	481±186* #	NA	NA
HOC+liver transplantation	1788±404	1560±166	1800±148*	1838±258*	2104±325*	2147±344*
hHGF-HOC+liver transplantation	1733±176	1558±248	2455±312	2664±206	3092±491	3512±228

Differences were analyzed using an analysis of variance or an independent sample *t*-test.

* *P*<0.05 liver transplantation *vs.* hHGF-HOC+liver transplantation or HOC+liver transplantation vs. hHGF-HOC+liver transplantation;

# P<0.05 liver transplantation vs. HOC+liver transplantation. NA, not available.

### Effect of HOC administration and transplantation on cytokine levels

**Figure 4:**
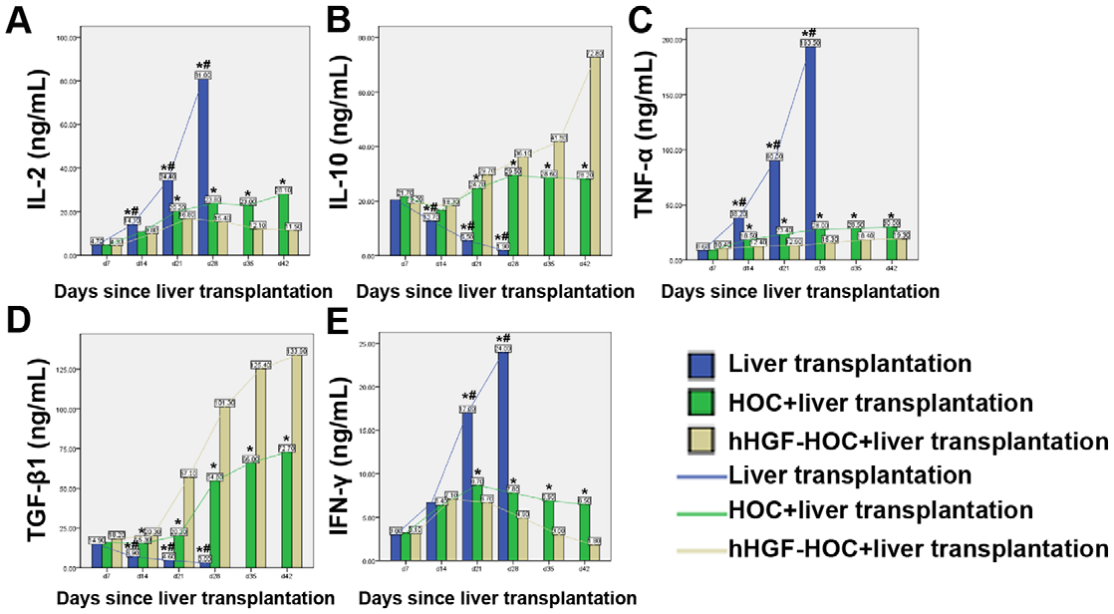
Analysis of serum cytokine levels post transplantation. Serum IL-2, IL-10, TNF-α, TGF-β1, and IFN-γ levels were examined at the indicated time points post transplantation by ELISA. Differences were analyzed using an analysis of variance or an independent sample *t*-test. *P<0.05, liver transplantation only vs. hHGF-HOC+liver transplantation or HOC+liver transplantation vs. hHGF-HOC+liver transplantation; #*P*<0.05, liver transplantation vs. HOC+liver transplantation.

We next investigated changes in serum T helper (Th) cells cytokine levels. Two weeks post surgery, transplantation in combination with the administration of HOC or hHGF-HOC significantly suppressed IL-2, TNF-α and IFN-γ production and enhanced the production of IL-10 and TGF-β1 compared to controls (*P*<0.05, Fig. 4). Furthermore, administration of hHGF-modified HOCs resulted in more significant changes to cytokine expression levels (*P*<0.05 compared to the HOC treated transplantation group).

### HOC administration prolongs survival of recipients

**Figure 5:**
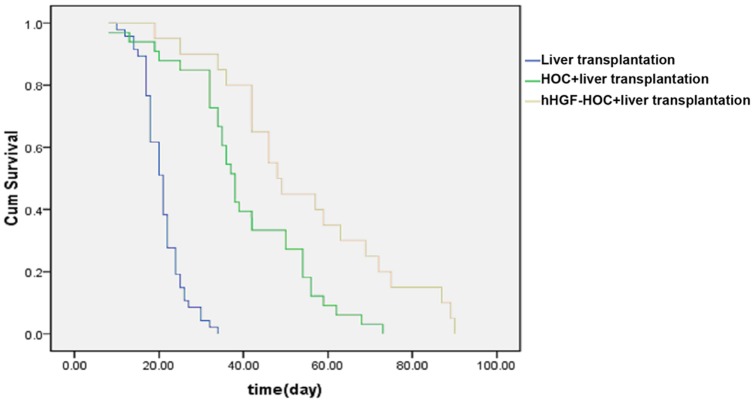
Survival curves of recipients.

Liver transplantation combined with the administration of either HOC or hHGF remarkably prolonged the median survival time of recipients compared to transplant control animals not treated with either HOC or hHGF (control, n = 47, median survival time = 21 days; HOC transplantation, n = 33, median survival time = 38 days; hHGF-HOC transplantation, n = 20, median survival time = 48 days; *P*<0.05). In addition, the median survival time of liver transplant rats treated with hHGF-HOC was significantly longer than that of HOC-treated liver transplant rats (*P*<0.05). As demonstrated by the recipient survival curve hHGF-HOC administration greatly prolonged animal survival rates compared to the other 2 treatment groups (Fig. 5).

### Transplantation combined with HOC treatment improved host liver function

Liver function indicators, ALT, DBil, ALB, GGT, ALP, and ChE were examined 1, 2, 3, 4, 5 or 6 weeks post transplantation (Table 2). HOC or hHGF-HOC treatment statistically down-regulated ALT levels 2 weeks post transplantation compared to controls (*P*<0.05). Compared to HOC treatment, administration of hHGF-HOC significantly reduced ALT levels 2 weeks post transplantation (*P*<0.05), suggesting that hHGF-HOC treatment prevented liver injury. hHGF-HOC treatment greatly decreased GGT and ALP values 2 weeks post transplantation and DBil levels 3 weeks post transplantation in liver transplant recipient rats (*P*<0.05 compared to HOC+liver transplantation or liver transplantation alone) suggesting that hHGF-HOC treatment promotes the function of intrahepatic bile ducts after transplantation. In addition, administration of hHGF-modified HOCs to transplant recipients also improved liver synthesis function (measured by increased ALB and ChE values) 2 and 3 weeks following transplantation (*P*<0.05 compared to HOC+liver transplantation or liver transplantation alone).

### Transplantation in the presence of HOCs promotes proliferation and decreased apoptosis

**Figure 6:**
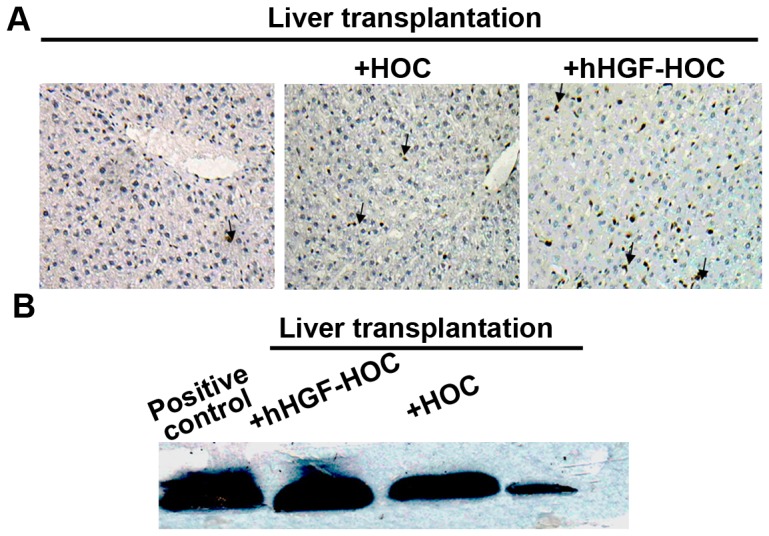
Cell proliferation and apoptosis. The beneficial effects of hHGF-HOCs on liver transplantation were evaluated by measuring cell proliferation and apoptosis. (A) Fourteen days post-transplantation, tissue sections were stained with specific antibodies to PCNA. Arrows indicated immuno-positive cells. Magnification ×100. (B) The protein expression of NFκB was examined by Western blot analysis.

Administration of HOCs or hHGF-modified HOCs to rats significantly promoted the number of proliferating cell nuclear antigen (PCNA)-positive cells 14 days post-transplantation (Fig. 6A). Compared to the HOCs group, rats transplanted with hHGF-HOCs transplantation had significantly elevated numbers of PCNA^+^ cells. NFκB (nuclear factor kappa-light-chain-enhancer of activated B cells) is an anti-apoptotic factor that regulates expression of a large number of genes critical to the regulation of apoptosis. We therefore examined NFκB expression levels in liver tissues 14 days following transplantation. Increased levels of NFκB were found in the HOCs and hHGF-HOCs groups compared to levels observed in controls (Fig. 6B). hHGF-HOCs significantly elevated the NFκB expression levels compared to levels observed in HOCs, suggesting that hHGF-HOC treatment may promote cell proliferation and decreases apoptosis following transplantation.

### Transplantation in the presence of HOCs decreased liver allograft rejection

**Figure 7:**
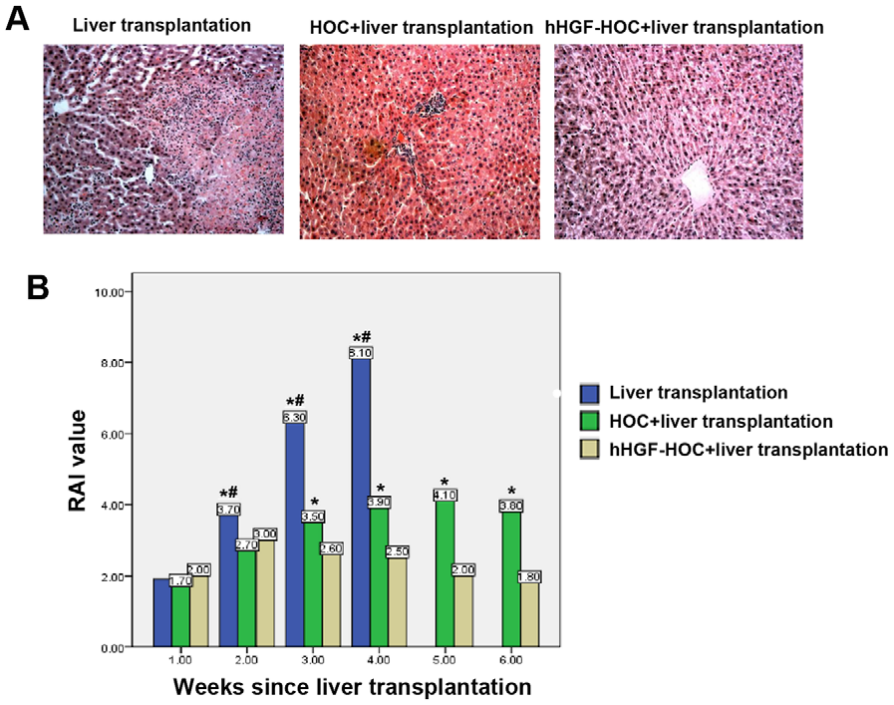
Histological examination of liver allograft rejection. (A) Sections obtained from experimental groups were staining with H&E, magnification ×100. (B) Liver allograft rejection was assessed using the rejection activity index according to the Banff classification of hepatic allograft rejection described in the Materials and Methods. Data were compared with analysis of variance or an independent sample *t*-test. *P<0.05 liver transplantation vs. hHGF-HOC+liver transplantation or HOC+liver transplantation vs. hHGF-HOC+liver transplantation; #P<0.05, liver transplantation vs. HOC+liver transplantation.

**Figure 8:**
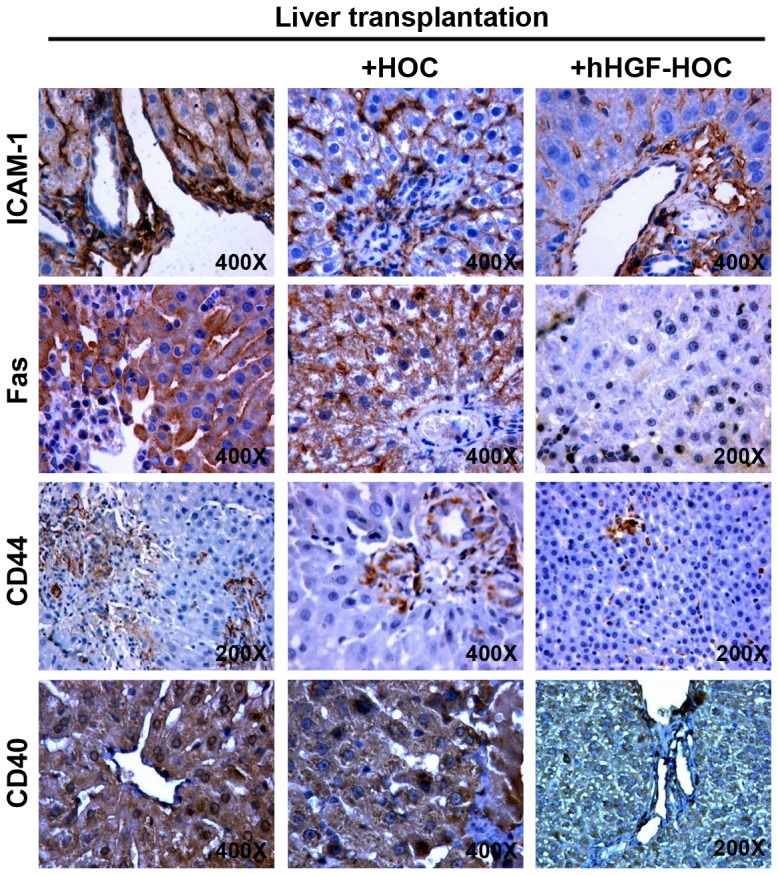
Immunostaining for ICAM-1, Fas, CD44 and CD40 28 days post transplantation. Immunopositive cells exhibited dark-brown staining. Sections were double stained with H&E (blue). Magnification is indicated on each image.

Administration of hHGF-modified HOCs to rats also receiving liver transplantation presented healthy liver tissues and significantly reduced the rejection activity index scores compared to control liver transplant rats or rats treated with HOCs that received a liver transplant (Fig. 7). In addition, the blood CD4^+^/CD8^+^ T lymphocyte ratio gradually increased in liver transplant rats with or without administration of HOC (Table 2). In contrast, transplantation in combination with hHGF-modified HOCs significantly decreased this ratio 2-weeks post transplantation (*P*<0.05 compared to the other groups). In addition, expression levels of ICAM-1, Fas, CD44, and CD40 in liver allografts were evaluated. ICAM-1 expression gradually increased in liver transplant recipient rats. Administration of HOCs or hHGF-modified HOCs greatly decreased ICAM-1 expression (percentage of sections with ICAM-1 positive staining 28 days following transplantation: liver transplantation, n = 55, 94.5%; HOC+liver transplantation, n = 53, 86.8%; hHGF-HOC+liver transplantation, n = 55, 81.8%; *P*<0.05 compared to liver transplantation) (Fig. 8). Combined hHGF-HOC transplantation significantly reduced the number of Fas-positive cells on day 28 post transplantation (liver transplantation, n = 55, 70.61±24.83 cells/mm^2^; HOC+liver transplantation, n = 53, 37.02±18.95 cells/mm^2^; hHGF-HOC+liver transplantation, n = 55, 12.80±7.54 cells/mm^2^; *P*<0.05 compared with liver transplantation only or HOC+liver transplantation). Similar results were obtained in the expression of CD44 and CD40. These results suggest that combined hHGF-HOC transplantation could decrease postoperative liver allograft rejection.

## Discussion

Even though liver transplantation is the most efficient means of treating patients with end-stage liver diseases, it is usually associated with postoperative complications, including acute allograft rejection [1], [2], chronic renal failure [14], post-transplantation lymphoproliferative disorders [15], and cardiovascular disease [16]. Among these side-effects, liver allograft rejection is considered to be a severe and major complication associated with significant morbidity and reduced life quality in liver transplant recipients [2]. Furthermore, the use of immunosuppressive agents may also cause a variety of adverse side effects such as inducing diabetes, hypertension, and nephrotoxicity, and therefore needs careful risk assessments before use [17]. Concomitant stem cell transplantation may induce allograft tolerance by modulating immune cell responses [18], [19]. In the present study, administration of HOC following liver transplantation significantly decreased acute liver rejection and prolonged the survival period of recipients.

Previous studies revealed that sustained expression of HGF could accelerate the proliferation of HOCs in a rat 2-AAF/PH model [10], [11], suggesting that modification to the HGF gene may promote *in vivo* proliferation and differentiation of HOCs, thereby benefitting liver regeneration post transplantation. In this study, a HOC line stably expressing the hHGF gene (hHGF-HOCs) was successfully established and shown *in vitro* to efficiently differentiate into large, round hepatocytes or into long spindle-like bile duct epithelial cells consistent with previous reports [10], [11].

Laboratory tests indicated that combined hHGF-HOC transplantation down-regulated ALT, DBil, GGT, ALP levels but up-regulated ChE, and ALB expression levels, suggesting that transplantation in combination with the administration of HOCs improved liver function, prevented bile duct damage, and protected against development of chronic hepatocyte injury. Since hHGF-modified HOCs are capable of secreting hHGF, it is possible that secreted hHGF may promote the proliferation, differentiation, and migration of HOCs in injured liver tissues while also enhancing the repair and regeneration of the donor liver. Most of the SRY-positive cells were observed surrounding the portal area, central vein, and bile duct. Therefore, it would be reasonable to suggest that these cells contributed to the protection of intrahepatic biliary epithelial cells.

The rat acute rejection orthotopic liver transplantation model [20] using DA rat livers transplanted into Lewis rats was used to assess the protective effects of HOC cell administration on acute liver rejection. Without interventions, liver recipients would die of acute rejection, postoperative infections, abdominal bleeding, or respiration and circulation failure within one week post surgery [20]. In this study, liver recipients were treated with tacrolimus 1 day prior to surgery until day 13 post surgery. Animals that died within 7 days post surgery were excluded from subsequent examinations.

Acute liver rejection can be triggered by immune responses mediated by different Th cell subtypes *via* redundant or synergistic pathways [21]. Th1 responses initiate allograft rejection by promoting cytotoxic T cell activitiy by producing IFN-γ, TNF-α, and IL-2 [21], [22]. By contrast, Th2 responses mediate allograft damage *via* eosinophil recruitment resulting from the production of IL-4, IL-5, IL-6, and IL-10 [22], [23]. Th1- and Th2-type cytokines regulate the Th1/Th2 paradigm during allograft responses [24], therefore, decreased levels of IFN-γ, TNF-α, and IL-2 accompanied by increased levels of IL-10 and TGF-β1 resulting from hHGF-HOC administration may reduce immunoreactivity and subsequently reduce acute liver rejection. Here, we observed a significantly decreased CD4^+^/CD8^+^ ratio in transplant rats also treated with hHGF-HOC, suggesting the induction of transplant tolerance. Furthermore, administration of hHGF-HOC to liver transplant rats significantly decreased expression of CD40, CD44, Fas and ICAM-1 (known bio-indicators for inflammatory cell infiltration and allograft rejection activity [25], [26], [27], [28]) compared to expression levels observed in other groups, suggesting that administration of hHGF-HOC to liver transplant recipients efficiently reduced inflammatory cell infiltration and subsequently decreased allograft rejection. In addition, we found that hHGF-HOCs promoted the number of PCNA positive cells while decreasing the expression of NFκB in liver tissues 14 days following transplantation, indicating that beneficial effects of hHGF-HOCs on liver transplantation might be due to increased cell proliferation and inhibition of apoptosis induced by hHGF-HOCs. Nevertheless, the underlying mechanism needs to be further defined.

In summary, our current study demonstrated that administration of HOCs stably expressing hHGF to liver transplant recipients significantly protected against acute allograft rejection and prolonged animal survival. Although the underlying molecular mechanism of hHGF-mediated prevention of acute liver rejection are unclear, it is possible that hHGF specifically binds to the c-Met tyrosine kinase receptor triggering downstream factors such as phosphatidylinositol 3-kinase (PI3K), phospholipase C (PLC)-γ and mitogen-activated protein kinase (MAPK) [8]. Future studies will be designed to investigate the role of signaling pathways associated with hHGF-mediated prevention of acute liver rejection.

## Materials and Methods

### Reagents

Fetal bovine serum (FBS) and phosphate buffered saline (PBS) were purchased from Invitrogen (Carlsbad, CA). DMEM (Dulbecco's Minimal Essential Media)/F12 culture medium was obtained from Sigma (St. Louis, MO). Hepatocyte growth factor (HGF), epidermal growth factor (EGF), stem cell factor (SCF) and leukemia inhibitory factor (LIF) were purchased from Millipore (USA). Plasmid pBLAST2-hHGF, blasticidin and lipofectamine 2000 transfection reagent were purchased from Invitrogen. The SRY *in situ* hybridization kit was purchased from the Tianjin Haoyang Biological Manufacture, Co., Ltd. (Tianjin, China). The hematoxylin and eosin (H&E) Staining Kit was purchased from the Shanghai Sangon Biological Engineering Technology and Services Co., Ltd. (Shanghai, China). ELISA kits for hHGF and rat IFN-γ, TGF-β1, TNF-α, IL-2, IL-10 were purchased from Shanghai ExCell Biology (Shanghai, China). Anti-rat CD3-FITC/CD4-PC7/CD8-APC antibodies were obtained from Beckman Coulter, Inc. (Brea, CA). Rat anti-CD44 monoclonal antibody and rat anti-ICAM-1 antibodies were purchased from Millipore (Billerica, MA). Rat anti-Fas and anti-CD40 antibodies were purchased from Stressgen Biotechnologies (San Diego, CA) and Santa Cruz Biotechnology (Santa Cruz, CA), respectively. Tacrolimus was purchased from Fujisawa Pharmaceutical Co. (Fujisawa, Japan).

### Animals

All animals were purchased from the Vital River Lab Animal Technology Co., Ltd. (Beijing, China) or the Laboratory Animal Center of Harbin Medical University Harbing, China). Male SPF Lewis rats (n = 10) weighing 200±20 g were used for HOC isolation. For transplantation, 180 female SPF Lewis rats (250–300 g in weight) and 180 female SPF dark agouti (DA) rats (200–350 g in weight) were used. All efforts were made to minimize animal suffering and to reduce the number of animals used. All animal procedures were approved by the Ethics Committee of Liaocheng People's Hospital.

### 2-AAF/PH model

Male Lewis rats (n = 10) were housed separately for one week prior to use. They were administered 20 mg/kg body weight 2-AAF dissolved in vegetable oil daily by intragastric administration for 6 days. On day 7, ten animals were partially hepatectomized (left lateral lobe excision, 1/2 to 1/3 PH) under general ether anesthesia without 2-AAF administration. 2-AAF feeding commenced on day 8 and the experiment concluded on day14.

### Primary HOC cultures

The 2-AAF/PH model was used to generate activated HOCs that were isolated 10 days post operation. Briefly, liver tissues were minced, washed with D-Hank's solution, and digested with 0.1% collagenase IV and 0.025% EDTA at 37°C. Cells were filtered through 100-mesh filters and centrifuged at 500 rpm for 5 min at 4°C. HOCs were separated by Percoll (Amersham Biosciences, Pittsburg, PA) gradient centrifugation. Cells were seeded onto gelatin-coated flasks and maintained in complete DMEM/F12 culture medium containing 10% FBS, 100 U/ml penicillin, 100 µg/ml streptomycin and amphotericin B (1 ng/ml) at 37°C in a 5% CO_2_ incubator. Three days after seeding, 20 ng/ml SCF, 10 ng/ml HGF, 10 ng/ml EGF and 10 ng/ml leukemia inhibitory factor (LIF) were added into the culture medium. To induce differentiation, media not containing LIF was added.

### Stable transfection of the pBLAST2-hHGF plasmid into HOCs

HOCs were transfected with plasmid pBLAST2-hHGF using the lipofectamine 2000 transfection reagent as previously described [29]. Briefly, for each 25 cm^2^ flask, 15 µl DNA was combined with 15 µl lipofectamine 2000 in 150 µl serum- and antibiotic-free DMEM/F12. After a 10–15 min incubation, the mixture was added to cells in the presence of 3 ml serum- and antibiotic-free DMEM/F12 culture medium. The cultures were incubated for 4 h in transfection medium that was then replaced with complete culture medium. Blasticidin-resistant cells were screened and maintained in DMEM/F12, 15% FBS, 20 ng/ml SCF, 10 ng/ml HGF, 10 ng/ml EGF, and 20 ng/ml LIF.

### Western blot analysis

Immunoblotting was performed as previously described [29]. Briefly, proteins were extracted from confluent cells using cell lysis buffer. Protein concentrations were determined using the Bradford assay and equal amounts of protein extracts were subjected to SDS-polyacrylamide gel electrophoresis, transferred to a polyvinylidene difluoride (PVDF) membrane, blocked with 5% skim milk in Tris-buffered saline (TBS), and then probed with anti-hHGF (1∶50 dilution) or anti-NFκB (1∶1000 dilution) at room temperature for 2 h. Secondary antibody was used at a dilution of 1∶1000 for 90 min at room temperature. Immunoblotting for β-actin was used as internal control. Proteins were transferred onto PVDF membranes, blocked with 5% skim milk in TBS containing 0.1% Tween-20 (TBST) at 37°C for 1 h, and then probed overnight with anti-β-actin (1∶3000 dilution) at 4°C. Goat anti-mouse HRP secondary antibody was used at a dilution of 1∶1000. Visualization of immunoreactive bands was carried out using an enhanced chemoluminescence detection method (ECL).

### Experimental design

The two-cuff (portal vein and infrahepatic vena cava) technique was used to establish orthotopic liver transplantation in rats as previously described [30]. Female DA rats were used as donors and female Lewis rats as recipients. Recipients were randomly divided into 3 groups: the control group (n = 60), HOC transplantation group (n = 60) and hHGF-modified HOC transplantation group (n = 60). Control group animals received orthotopic liver transplantation and transplantation group animals received either 1 ml of 1×10^6^ cells/ml HOCs or hHGF-modified HOCs (passage 4–10) injected into their portal veins and hepatic arteries during orthotopic liver transplantation. Recipients were given tacrolimus one day prior to surgery until 13 days post operation.

### Liver function assessment

Liver function indicators, including measurement of alanine aminotransferase (ALT), direct bilirubin (DBil), albumin (ALB), γ-glutamyltransferase (GGT), alkaline phosphate (ALP), and cholinesterase (ChE) levels were determined at the indicated time points after transplantation using a Beckman CX9 automatic biochemical analyzer (Germany).

### *In situ* hybridization

Donor tissues post transplantation were carefully removed and fixed in 10% buffered formaldehyde. Paraffin-embedded sections were rehydrated using a graded ethanol series (95, 80, 60, 30%) with 3–5 min hydration incubations. After a 10 min incubation with 3% H_2_O_2_ at room temperature, sections were incubated with complex digestive solution for another 10 min at 37°C. After washing with Tris-buffered saline (TBS) and 0.2× SSC, samples were further incubated with the probe working solution at 37°C for 4–8 h. Then, sections were treated with peroxidase (POD) followed by DAB solution, and then stained with H&E. At least 10 images were randomly captured using a phase contrast microscope (Olympus, Japan).

### Quantitative RT-PCR

Total RNA was extracted from HOCs, differentiated HOCs, liver tissues, or bile duct tissues using Trizol reagent (Sigma-Aldrich, USA). After purification, the integrity of the purified mRNA was confirmed by agarose gel electrophoresis. The cDNA was then transcribed using the First cDNA Synthesis Kit according to the manufacturer's instructions (Fermentas, CA, USA). The sequences of the specific primers used were as follows: ALB forward primer, 5′-TGTCACGGCGACCTGTTG-3′, reverse primer, 5′-GGAGATAGTGGCCTGGTTCTCA-3′; CK19 forward primer, 5′-GACTTCCGGACCAAGTTTGAG-3′, reverse primer 5′-CGCAGGCCGTTGATGTC-3′; and β-actin forward primer, 5′-ACCGAGCGCGGCTACAGC-3′ and reverse primer, 5′-CTCATTGCCAATGGTGAT-3′. PCR amplification was carried out on a real-time fluorescence quantitative instrument (Roche Diagnostics, Switzerland). The thermocycling conditions were as follows: 60°C for 2 min, 94°C for 10 min, and then 40 cycles at 94°C for 15 s and 60°C for 60 s. The amplified products were analyzed with real-time fluorescence quantitative instrument software. Fold changes in target gene expression were then normalized using the following formula: Fold change = 2^−Δ(ΔCt)^. The negative log was calculated and data were analyzed from 20 independent experiments.

### Immunohistochemical analysis

Sections were fixed with 4% paraformaldehyde (PFA), dewaxed in xylene, subjected to a graded ethanol series, rinsed with PBS, treated with 3% H_2_O_2_ and citrate solution, and immunostained with primary antibodies specific for ALB, CK19, PCNA, CD44, Fas, ICAM-1, or CD40 at 37°C for 2 h or at 4°C overnight. After washing with PBS 3 times, sections were stained with goat anti-rabbit biotinylated secondary antibody for 20 min at 37°C. After PBS washing, samples were incubated with SABC solution followed by DAB solution. After double staining with H&E, samples were mounted and fluorescence was detected using a fluorescence microscope (Olympus, Japan).

### Evaluation of liver allograft rejection

The severity of acute rejection was assessed using the rejection activity index according to the Banff classification of hepatic allograft rejection [31]. For portal inflammation, 1 = primarily lymphocytic inflammation involving but not noticeably expanding to a minority of the portal triads, 2 = expansion of infiltrates containing a mixture of lymphocytes with occasional blasts, neutrophils and eosinophils into most or all of the portal triad, and 3 = marked expansion of mixed infiltrates containing numerous blasts and eosinophils into most or all of the portal triads with inflammatory spillover into the periportal parenchyma.

For bile duct inflammation damage, 1 = a minority of the ducts are cuffed presenting with inflammatory cells and show only mild reactive changes, and 2 = most or all of the ducts contain inflammatory cell infiltrates. More than an occasional duct shows degenerative changes.

For venous endothelial inflammation, 1 = subendothelial lymphocytic infiltration involving some, but not a majority of the portal and/or hepatic venules, 2 = subendothelial infiltration involving most or all of the portal and/or hepatic venules, 3 = similar to 2 but with moderate to severe perivenular inflammation that extends into the perivenular parenchyma and is associated with perivenular hepatocyte necrosis. The total score is graded as follows: 0–2, no rejection; 3, borderline; 4–5, mild rejection; 6–7, moderate rejection; 8–9, severe rejection.

### ELISA

hHGF and cytokine levels were evaluated by ELISA. Primary and secondary antibodies were diluted 1∶250 with antibody dilution solution. Biotinylated secondary antibody was added to the 96-well plate wells (100 µl/well) and incubated with samples at 37°C for 90 min. Then, 100 µl of enzyme solution was added to each well and the plate incubated at 37°C for 30 min. After washing, 100 µl of substrate solution was added and plates incubated at 37°C for 15 min in the dark. Stop solution was added (100 µl/well) and the absorbance at 450 nm measured using a plate reader (BioTek, Winooski, VT) immediately after complete mixing.

### CD3/CD4/CD8 lymphocyte counting

Anti-rat CD3-FITC/CD4-PC7/CD8-APC antibodies were mixed with the same volume of blood. After a 20 min incubation in the dark at room temperature, lysis buffer was added and percentages of positive cells analyzed using FACS (BD Bioscience).

### Statistical analysis

Data were analyzed using SPSS 16.0 software and the data were expressed as the mean ± SD. Comparison of median survival time was carried out using Kaplan-Meier survival analysis. Differences in data measurements were compared with analysis of variance or an independent sample *t*-test. *P*<0.05 was considered as statistically different.

## References

[1] Kaufman DB, Shapiro R, Lucey MR, Cherikh WS, R TB, et al (2004) Immunosuppression: practice and trends. Am J Transplant 4 Suppl 9: 38–53. 15113354 10.1111/j.1600-6135.2004.00397.x

[2] Knechtle SJ, Kwun J (2009) Unique aspects of rejection and tolerance in liver transplantation. Semin Liver Dis 29: 91–101. 19235662 10.1055/s-0029-1192058

[3] Tosh D, Strain A (2005) Liver stem cells–prospects for clinical use. J Hepatol 42 Suppl: S75–84. 15777575 10.1016/j.jhep.2004.12.009

[4] Faris RA, Konkin T, Halpert G (2001) Liver stem cells: a potential source of hepatocytes for the treatment of human liver disease. Artif Organs 25: 513–521. 11493271 10.1046/j.1525-1594.2001.025007513.x

[5] Fausto N, Campbell JS (2003) The role of hepatocytes and oval cells in liver regeneration and repopulation. Mech Dev 120: 117–130. 12490302 10.1016/s0925-4773(02)00338-6

[6] Smets F, Najimi M, Sokal EM (2008) Cell transplantation in the treatment of liver diseases. Pediatr Transplant 12: 6–13. 18186884 10.1111/j.1399-3046.2007.00788.x

[7] Matsumoto K, Nakamura T (1996) Emerging multipotent aspects of hepatocyte growth factor. J Biochem 119: 591–600. 8743556 10.1093/oxfordjournals.jbchem.a021283

[8] Huh CG, Factor VM, Sanchez A, Uchida K, Conner EA, et al (2004) Hepatocyte growth factor/c-met signaling pathway is required for efficient liver regeneration and repair. Proc Natl Acad Sci U S A 101: 4477–4482. 15070743 10.1073/pnas.0306068101 PMC384772

[9] Kato H, Shimomura T, Murai R, Gonda K, Ishii K, et al (2007) Regulation of hepatic oval cell proliferation by adenoviral mediated hepatocyte growth factor gene transfer and signal transduction inhibitors. Hepatogastroenterology 54: 821–825. 17591071

[10] Hasuike S, Ido A, Uto H, Moriuchi A, Tahara Y, et al (2005) Hepatocyte growth factor accelerates the proliferation of hepatic oval cells and possibly promotes the differentiation in a 2-acetylaminofluorene/partial hepatectomy model in rats. J Gastroenterol Hepatol 20: 1753–1761. 16246197 10.1111/j.1440-1746.2005.03922.x

[11] Shiota G, Kunisada T, Oyama K, Udagawa A, Nomi T, et al (2000) In vivo transfer of hepatocyte growth factor gene accelerates proliferation of hepatic oval cells in a 2-acetylaminofluorene/partial hepatectomy model in rats. FEBS Lett 470: 325–330. 10745090 10.1016/s0014-5793(00)01337-5

[12] Azuma H, Takahara S, Matsumoto K, Ichimaru N, Wang JD, et al (2001) Hepatocyte growth factor prevents the development of chronic allograft nephropathy in rats. J Am Soc Nephrol 12: 1280–1292. 11373353 10.1681/ASN.V1261280

[13] Yamaura K, Ito K, Tsukioka K, Wada Y, Makiuchi A, et al (2004) Suppression of acute and chronic rejection by hepatocyte growth factor in a murine model of cardiac transplantation: induction of tolerance and prevention of cardiac allograft vasculopathy. Circulation 110: 1650–1657. 15364799 10.1161/01.CIR.0000143052.45956.71

[14] Chung H, Kim KH, Kim JG, Lee SY, Yoon YH (2007) Retinal complications in patients with solid organ or bone marrow transplantations. Transplantation 83: 694–699. 17414700 10.1097/01.tp.0000259386.59375.8a

[15] Patel H, Vogl DT, Aqui N, Shaked A, Olthoff K, et al (2007) Posttransplant lymphoproliferative disorder in adult liver transplant recipients: a report of seventeen cases. Leuk Lymphoma 48: 885–891. 17487731 10.1080/10428190701223275

[16] Tamsel S, Demirpolat G, Killi R, Aydin U, Kilic M, et al (2007) Vascular complications after liver transplantation: evaluation with Doppler US. Abdom Imaging 32: 339–347. 16967253 10.1007/s00261-006-9041-z

[17] Mukherjee S, Mukherjee U (2009) A comprehensive review of immunosuppression used for liver transplantation. J Transplant 2009: 701464. 20130772 10.1155/2009/701464 PMC2809333

[18] Aggarwal S, Pittenger MF (2005) Human mesenchymal stem cells modulate allogeneic immune cell responses. Blood 105: 1815–1822. 15494428 10.1182/blood-2004-04-1559

[19] Locke JE, Shamblott MJ, Cameron AM (2009) Stem cells and the liver: clinical applications in transplantation. Adv Surg 43: 35–51. 19845168 10.1016/j.yasu.2009.03.002

[20] Zimmermann FA, Knoll PP, Davies HS, Gokel JM, Schmid T (1983) The fate of orthotopic liver allografts in different rat strain combinations. Transplant Proc 25: 1272. 10.1097/00007890-198404000-00019 6369673

[21] Mosmann TR, Coffman RL (1989) TH1 and TH2 cells: different patterns of lymphokine secretion lead to different functional properties. Annu Rev Immunol 7: 145–173. 2523712 10.1146/annurev.iy.07.040189.001045

[22] Chang JT, Reiner SL (2008) Specifying helper T cell fates during immunity. J Pediatr Gastroenterol Nutr 46 Suppl 1: E19–20. 18354322 10.1097/01.mpg.0000313832.47207.0e

[23] VanBuskirk AM, Wakely ME, Orosz CG (1996) Transfusion of polarized TH2-like cell populations into SCID mouse cardiac allograft recipients results in acute allograft rejection. Transplantation 62: 229–238. 8755821 10.1097/00007890-199607270-00014

[24] Strom TB, Roy-Chaudhury P, Manfro R, Zheng XX, Nickerson PW, et al (1996) The Th1/Th2 paradigm and the allograft response. Curr Opin Immunol 8: 688–693. 8902395 10.1016/s0952-7915(96)80087-2

[25] Marhaba R, Zoller M (2004) CD44 in cancer progression: adhesion, migration and growth regulation. J Mol Histol 35: 211–231. 15339042 10.1023/b:hijo.0000032354.94213.69

[26] Hubbard AK, Rothlein R (2000) Intercellular adhesion molecule-1 (ICAM-1) expression and cell signaling cascades. Free Radic Biol Med 28: 1379–1386. 10924857 10.1016/s0891-5849(00)00223-9

[27] Strasser A, Jost PJ, Nagata S (2009) The many roles of FAS receptor signaling in the immune system. Immunity 30: 180–192. 19239902 10.1016/j.immuni.2009.01.001 PMC2956119

[28] Chatzigeorgiou A, Lyberi M, Chatzilymperis G, Nezos A, Kamper E (2009) CD40/CD40L signaling and its implication in health and disease. Biofactors 35: 474–483. 19904719 10.1002/biof.62

[29] Yang Y, Fukui K, Koike T, Zheng X (2007) Induction of autophagy in neurite degeneration of mouse superior cervical ganglion neurons. Eur J Neurosci 26: 2979–2988. 18001292 10.1111/j.1460-9568.2007.05914.x

[30] Xie R, Xu SR (2006) Two-cuff technique was applied in the establishment of orthotopic liver transplantation in rats. Journal of Jiangsu University (Medicine Edition) 16: 392–395.

[31] Solez K (2010) History of the Banff classification of allograft pathology as it approaches its 20th year. Curr Opin Organ Transplant 15: 49–51. 19949334 10.1097/MOT.0b013e328334fedb

